# IMRT credentialing for prospective trials using institutional virtual phantoms: results of a joint European Organization for the Research and Treatment of Cancer and Radiological Physics Center project

**DOI:** 10.1186/1748-717X-9-123

**Published:** 2014-05-29

**Authors:** Damien C Weber, Veronique Vallet, Andrea Molineu, Christos Melidis, Vanda Teglas, Suzanne Naudy, Raphael Moeckli, David S Followill, Coen W Hurkmans

**Affiliations:** 1 Center for Proton Therapy, Paul Scherrer Institute, Villigen CH-5232, Switzerland; 2 University of Bern, Bern, Switzerland; 3 EORTC QA Strategic Committee-ROG, Brussels, Belgium; 4 Institute of Radiation Physics, Lausanne University Hospital, Lausanne, Switzerland; 5 Department of Radiation Physics, The University of Texas M. D. Anderson Cancer Center, Houston, TX 77030, USA; 6 EORTC Headquarter QA team, Brussels, Belgium; 7 Centre Georges-François-Leclerc, Dijon, France; 8 Department of Radiation Oncology, Catharina Hospital, Eindhoven, The Netherlands

**Keywords:** IMRT credentialing, Prospective clinical trial, Virtual phantom, Anthropomorphic phantom, Quality assurance

## Abstract

**Background and purpose:**

Intensity-modulated radiotherapy (IMRT) credentialing for a EORTC study was performed using an anthropomorphic head phantom from the Radiological Physics Center (RPC; RPC_PH_). Institutions were retrospectively requested to irradiate their institutional phantom (INST_PH_) using the same treatment plan in the framework of a Virtual Phantom Project (VPP) for IMRT credentialing.

**Materials and methods:**

CT data set of the institutional phantom and measured 2D dose matrices were requested from centers and sent to a dedicated secure EORTC uploader. Data from the RPC_PH_ and INST_PH_ were thereafter centrally analyzed and inter-compared by the QA team using commercially available software (RIT; ver.5.2; Colorado Springs, USA).

**Results:**

Eighteen institutions participated to the VPP. The measurements of 6 (33%) institutions could not be analyzed centrally. All other centers passed both the VPP and the RPC ±7%/4 mm credentialing criteria. At the 5%/5 mm gamma criteria (90% of pixels passing), 11(92%) as compared to 12 (100%) centers pass the credentialing process with RPC_PH_ and INST_PH_ (p = 0.29), respectively. The corresponding pass rate for the 3%/3 mm gamma criteria (90% of pixels passing) was 2 (17%) and 9 (75%; p = 0.01), respectively.

**Conclusions:**

IMRT dosimetry gamma evaluations in a single plane for a H&N prospective trial using the INST_PH_ measurements showed agreement at the gamma index criteria of ±5%/5 mm (90% of pixels passing) for a small number of VPP measurements. Using more stringent, criteria, the RPC_PH_ and INST_PH_ comparison showed disagreement. More data is warranted and urgently required within the framework of prospective studies.

## Introduction

The delivery of radiation therapy (RT) to Head and Neck (H&N) cancers is highly complex and challenging in terms of tumour delineation and treatment delivery. Recognizing the complexity of these treatments, especially delivered with Intensity Modulated RT (IMRT) in a multi-institutional clinical trial setting, the National Cancer Institute (NCI) funded clinical trial groups established an IMRT credentialing program administered by the Radiological Physics Center (RPC). Up to 2011, the RPC has evaluated 1139 anthropomorphic Head and Neck (H&N) IMRT phantom irradiations as a part of its credentialing program for participation in NCI funded clinical trials that allow the use of IMRT [1]. Early in the credentialing process, it was reported that nearly 28% of the institutions could not pass this irradiation study on their first attempt with generous acceptance criteria of ±7% for dose in a low gradient and/or ±4 mm distance to agreement in high gradient region [2]. More recently, a follow-up publication has shown that the pass rate has increased to nearly 82% indicating an improved implementation of IMRT over the past 10 years [3]. Even with these improvements, it is of interest to note that 18% of irradiations did not pass this phantom study. Molineu et al. also reported that after 12 years of credentialing, an analysis of the irradiations showed that the credentialing failure rate doubled when the criteria was reduced from ±7%/4 mm to ± 5%/4 mm [3]. As such, delivering advanced technology RT for H&N cancers in clinical trials remains challenging and requires RT credentialing before a facility is allowed to participate in a specific clinical trial.

Conducting clinical trial research in the cooperative group setting requires a set of defined standards and consistent treatments for investigator and site participation in order for the trial results to be valid and extendable to the broader oncology community. Even though the benefits of QA and RT credentialing have been demonstrated [4-6], there can be resistance to credentialing requirements as they are sometimes perceived to be an unnecessary burden, simply due to the extra effort needed to perform the credentialing requirements. Despite an institution’s concern that credentialing requirements, that may include a phantom irradiation study, completion of a questionnaire or having the treatment plan approved prior to commencing the RT treatment, are burdensome, this extra burden has been shown to reduce deviations by minimizing the patient data uncertainty used to analyze the trial outcomes. There are many ways to credential institutions to participate in clinical trials and different methods should be investigated to find improved and more efficient processes. We undertook a study to determine whether the use of an institution’s own phantom and QA measurements analyzed centrally by a dedicated QA team could be correlated with the end-to-end anthropomorphic phantom irradiation results for Complex Dosimetry Checks (CDC).

The QA Strategic Committee from the European Organization for the Research and Treatment of Cancer (EORTC) thus decided to compare the measured dose distributions obtained using the anthropomorphic IMRT H&N phantom irradiation results from the RPC for a new H&N EORTC study (22071-24071) to each institution’s own IMRT QA measurements obtained using their pre-treatment verification methodology. This paper presents a comparison of the RPC’s measured planar dose distribution results and the institutional phantom IMRT QA measurement results for the EORTC Virtual Phantom Project (VPP). This work is presented as collaboration between these two QA centers in the framework of the Global Harmonization Group, which thrive to harmonize and improve the quality assurance of radiation therapy implemented worldwide ( http://www.rtqaharmonisation.org/).

## Patients and methods

The planned QA activities of a non-activated randomized phase III H&N trial, assessing postoperative chemoradiation in combination with anti EGFR-antibody *vs*. postoperative chemoradiation in squamous cell carcinomas (EORTC 22071-24071), included a facility questionnaire, an External Reference Dosimetry Audit (ERDA), a Dummy Run, Individual Case reviews and CDCs for IMRT (i.e. QART level 5 [7]). This phase III study was approved by institutional ethics committee of all participating centers (Additional file 1). Prior to an institution being allowed to use IMRT to treat patients on this trial it had to successfully complete an IMRT CDC in accordance with the QART protocol guidelines. As such, 18 EORTC centers participated in the IMRT credentialing process before their activation to the trial.

**Figure 1:**
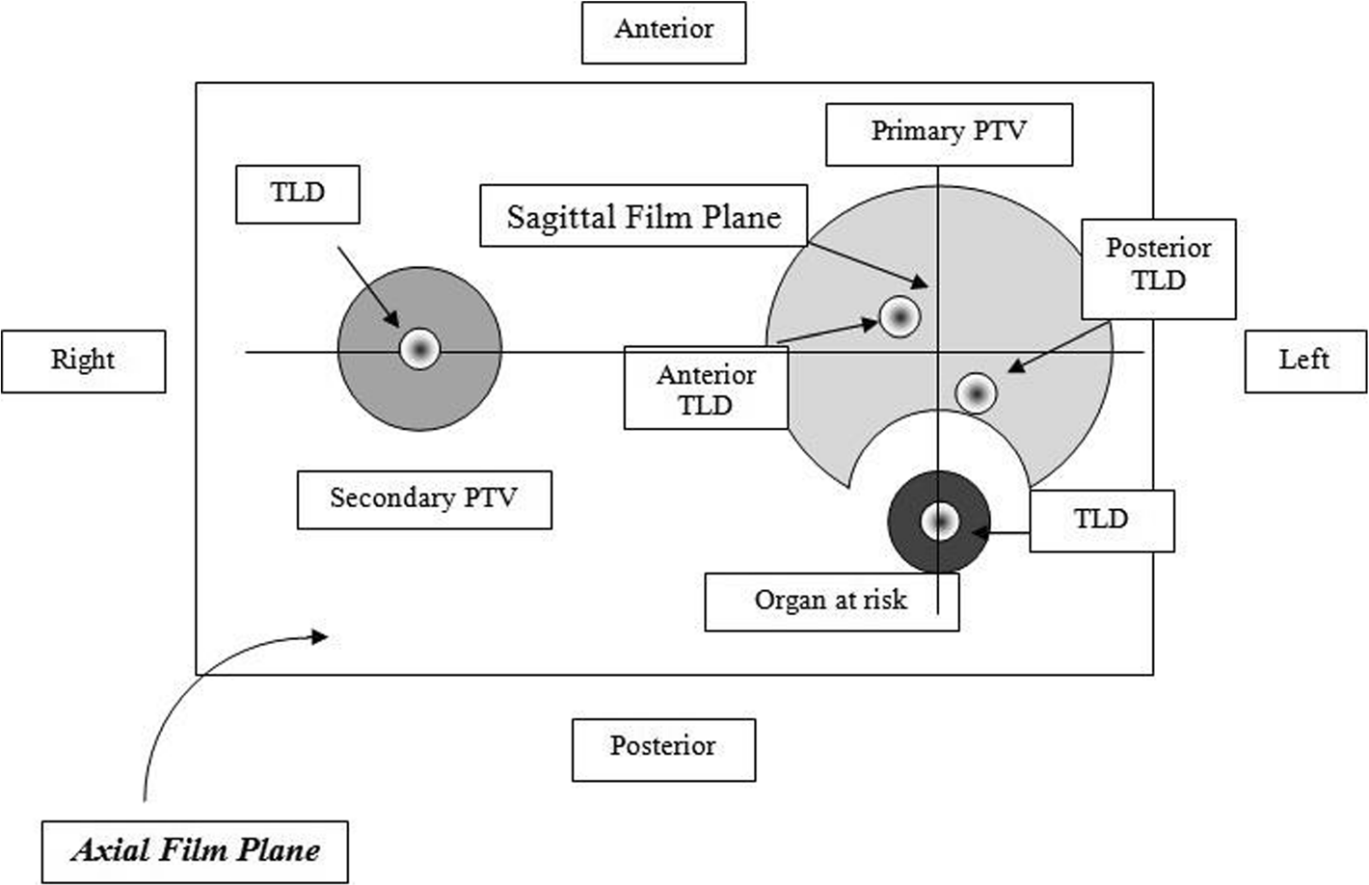
Cross-sectional view of the insert in the axial film plane.

This study compares some of the dosimetry measurements obtained as a part of the CDCs performed by the RPC (Houston, USA; http://rpc.mdanderson.org) using their IMRT H&N anthropomorphic phantom (RPC_PH_ CDC) and dosimetry measurements performed by each institution using their own IMRT QA phantom and plan from the RPC phantom irradiation (INST_PH_ CDC). The RPC_PH_ CDC’s phantom dosimetry insert contains eight Thermo Luminescent Dosimeters (TLDs) within the primary and secondary PTVs and one organ at risk (OAR) (Figure 1). The phantom has two radiochromic films going through the primary PTV in the axial and sagital planes. Only the RPC_PH_ CDC measured dose distribution data in the sagital plane were used for comparison in this study. The INST_PH_ CDCs used in the analyses included 2D dose measurements in coronal and/or sagital planes as supplied by the institutions. All dosimetry measurements, RPC_PH_ and INST_PH_ CDCs, were collected and analyzed as absolute dose utilizing absolute point dose measurements performed for each CDC. The RPC_PH_ CDC normalization was performed using the measured TLD doses whereas the INST_PH_ CDC normalization was to the dose distribution’s maximum dose. All INST_PH_ CDCs beams were measured using the gantry angle as defined in the clinical treatment plan and all, except one, were measurements of all fields combined. All measurement data were centrally collected, compared and analyzed at the EORTC’s QA center.

Dose distributions were compared using the gamma index function described by Low et al. [8], normalized using the predicted dose. The standard RPC_PH_ CDC analysis used an acceptance criterion of ±7%/4 mm criteria established in conjunction with clinical trial groups for both the axial and sagital film planes. For this study, the RPC provided 2D gamma analyses to the EORTC, using ±3%/3 mm, ±5%/5 mm and ±7%/4 mm criteria, for 18 phantom irradiations. The gamma evaluation was performed using a global comparison method and a prescription dose of 6.6 Gy (RPC’s dose level for CDC) to which the dose was normalized. The dose distribution area evaluated was a rectangular film area encompassing the primary PTV and OAR.

For the INST_PH_ CDCs, each EORTC institution was asked to CT scan their in-house IMRT QA phantom and recalculate a hybrid treatment plan dose distribution for their INST_PH_ CDC, using the same treatment planning system (TPS), monitor units and fields as used for the RPC phantom irradiation. The number of planes and point doses evaluated for each institution varied between multiple planes of data being provided to a single plane of data being used for this analysis. The various different TPSs and INST_PH_ CDC measurement systems used in this VPP are shown in Table 1. The INST_PH_ CDC was performed at each of the EORTC institutions and one or more 2D dose distributions were generated. The TPS DICOM-RT datasets of the INST_PH_ CDC, and the institution’s IMRT QA measurement system datasets were collected and compressed into one file and sent to the EORTC virtual QA platform ( https://uploader.eortc.be/qat/). Using the institution’s TPS and dosimetry QA measurement data, the institution’s TPS and QA measurement comparisons were performed by the EORTC QA team using the Radiological Imaging Technology software 113 (RIT; ver.5.2; Colorado Springs, USA). The RPC analysis used an in-house software with a rectangular area of interest while EORTC used the RIT113 software from RIT disregarding the dose below 5% of the prescribed dose.

**Table 1:** Institutional treatment planning systems and anthropomorphic quality assurance phantoms performing the VPP for 12 EORTC centers

**Treatment planning systems**	**Number (%)**	**Type of computational algorithms**
Eclipse	7 (58.3)	AAA
Tomotherapy	2 (16.7)	CSA
Pinnacle	2 (16.7)	CC
Monaco	1 (8.3)	Monte Carlo
**Type of quality assurance phantom and measurement tool**	**Number (%)**	**Registration method**
Delta4	4 (33.3)	Central
Cheese fantom + film	1 (8.3)	Automatic
Octavius + 2D-array	4 (33.3)	Central
Portal Dosimetry	2 (16.7)	Central
Solid water slabs + film	1 (8.3)	Automatic

*Abbreviations*: CSA/AAA: Convolution superposition algorithm-Analytical anisotropic algorithm; CC: Collapsed Cone algorithm.

The calculated and measured INST_PH_ CDC dose distributions were compared using a global dose gamma index evaluation with ±3%/3 mm, ±5%/5 mm and ±7%/4 mm criteria and the maximum measured dose as the normalization point (this absolute dose was considered as the 100% dose in the gamma analysis) instead of the prescription dose as used in the RPC analysis. The participating institutions provided their film dosimetry calibration curve to correctly convert grey-scale to dose. No rescaling of the calculated dose distributions was performed whereas the RPC measured dose distributions were rescaled to the measured TLD dose in the primary PTV. Institutions used the Delta4, 2D-array and portal device as absolute dose measurement tools (Table 2).

**Table 2:** RPC and VPP average percentage of pixels passing the gamma criteria at 3%/3 mm, 5%/5 mm and 7%/4 mm for 12 institutions of the EORTC for IMRT complex dosimetry checks

	**RPC**					**VPP**		
**Institution number**	**3%/3 mm (%) RPC**_**PH**_	**5%/5 mm (%) RPC**_**PH**_	**7%/4 mm (%) RPC**_**PH**_	**Number of verified fields**	**Measurement device**	**3%/3 mm (%) [SD] INST**_**PH**_	**5%/5 mm (%) [SD] INST**_**PH**_	**7%/4 mm (%) [SD] INST**_**PH**_
1	89.3	99.9	99.8	Combined	Delta4	100.0 [-]	100.0 [-]	100.0 [-]
2	62.9	82.0	91.2	Combined	PTW 2D-array	93.2 [-]	99.0 [-]	99.4 [-]
3	78.1	99.9	97.8	Combined	PTW 2D-array	90.8 [0.9]	100 [0.2]	100 [0.3]
4	84.7	98.2	99.5	Combined^a^	PTW 2D-array	84.1 [1.9]	95.7 [0.6]	96.4 [0.7]
5	80.1	98.2	99.3	Combined^a^	Varian Portal	96.5 [3.3]	99.8 [0.3]	100.0 [0.0]
6	96.4	99.8	99.9	9	Varian Portal	96.6 [2.2]	99.4 [1.3]	100.0 [0.0]
7	96.5	99.8	100.0	Combined	PTW 2D-array	85 [1.3]	96.6 [0.6]	97.3 [0.5]
8	89.1	96.9	98.1	Combined	EDR2-Film	89.1 [-]	99.3 [-]	99.9 [-]
9	73.5	91.9	95.5	Combined	Delta4	99.5 [-]	100.0 [-]	100.0 [-]
10	79.6	94.1	98.8	Combined	Delta4	98.4 [-]	100.0 [-]	100.0 [-]
11	89.9	98.9	99.7	Combined	EDR2-Film	92.2 [-]	97.0 [-]	100.0 [-]
12	72.5	93.0	96.8	Combined	Delta4	97.1 [-]	100.0 [-]	100.0 [-]
CDC								
Pass at 80% pixel level	7	12	12			12	12	12
Pass at 90% pixel level	2	11	12			9	12	12
Pass at 95% pixel level	2	8	11			6	12	12

*Abbreviations*: RPC: Radiological Physics Center; VPP: Virtual Phantom Project; SD: Standard Deviation; CDC, complex dosimetry checks. Combined: A combined measurement of all fields is made ^a^: measured twice.

Except for the institution film dosimetry, the dose distributions were registered using the "central registration" option as the isocenter corresponded to the center of the measurement device. Due to lack of usable reference points an automatic registration option based on best guess was used for film dosimetry that may have introduced some rotational uncertainty in the registration of the two dose distributions, but is not anticipated to significantly influence the results.

RPC and VPP gamma indexes were compared using the Wilcoxon signed ranks test. Differences were regarded as statistically significant at the p < 0.05 level. Analyses were performed on the Statistical Package for Social Sciences (SPSS, Ver. 18.0, SPSS Inc., Chicago, IL).

## Results

### RPC_PH_ CDC phantom study

Table 2 details the RPC dose analyses for 12 phantom irradiations. In total, 36 RPC dose distribution gamma index evaluations were calculated for this study and compared to the dose distributions gamma evaluations from the 12 EORTC institutions that provided their IMRT data using ±3%/3 mm, ±5%/5 mm and ±7%/4 mm at 80%, 90% and 95% pixel pass rates for each criteria level. Ten (83%) and 1 (8%) of the 12 RPC phantom irradiations, using the sagital film plane only, did not pass the gamma criteria of ±3%/3 mm and ±5%/5 mm (90% of the pixels meeting the criteria), respectively. None of the films in the 12 irradiations failed the RPC gamma criteria of ±7%/4 mm (at either the 80% (current) or 95% of pixels passing) as noted in Table 2. Table 3 details the RPC gamma evaluation of the sagittal plane dose distribution and the institution’s VPP gamma results (represented as the average percent of pixels passing each defined criteria).

**Table 3:** Wilcoxon signed ranks test of the RPC sagital film plane and VPP gamma indexes mean values

	**3%/3 mm (%) [SD]**	***p *** **value**	**5%/5 mm (%) [SD]**	***p *** **value**	**7%/4 mm (%) [SD]**	***p *** **value**
		0.02		0.21		0.08
RPC (RPC_PH_)	82.7 [10.2]		96.1 [5.3]		98.0 [2.6]	
VPP (INST_PH)_	93.5 [5.4]		98.9 [1.5]		99.4 [1.2]	

*Abbreviations*: RPC: Radiological Physics Center; VPP: Virtual Phantom Project.

### INST_PH_ CDC study

An INST_PH_ CDC was performed by 12 centers (Table 2). Table 2 details the average dose distribution gamma evaluation percent of pixels meeting the 3 different criteria levels for the multiple planes of data provided by each institution to the EORTC QA team representing the multiple gantry angles for the entire dose delivery. The IMRT QA measurements from six out of 18 institutions (33%) could not be analyzed centrally at the EORTC for the following reasons. Downloaded files (in .opd, .asc or .dcm format) from 2 centers could not be read by the RIT software, a header file from a Tomotherapy data file was missing in 2 other centers, missing files with the scanned measurement file uploaded to the digital platform in another center and finally a missing .opg MatriXX measurement file that could not be uploaded in an additional center. The inability to receive and analyze the institution’s TPS and dosimetry data for a third of the institutions warrants further investigation and an improved data receipt method before the VPP can be implemented.

In total, 20 INST_PH_ dose maps from 12 EORTC institutions were analyzed using gamma QA criteria of ±3%/3 mm, ±5%/5 mm and ±7%/4 mm (Table 2). Nine (75%) of the 12 centers met the more stringent 3%/3 mm (90% of pixels passing) gamma criteria that is commonly used by institutions to perform their own IMRT QA as compared to only 17% (2/12) of the RPC phantom irradiations. Likewise, 12 (100%) of the 12 EORTC centers as compared to 11 (92%) of the RPC phantom irradiation met the 5%/5 mm gamma criteria (90% of pixels passing).

### RPC and VPP institutional dosimetry comparison

The RPC and institution VPP dosimetry measurements for the two datasets were compared (Table 3). Using the current RPC gamma criteria ±7%/4 mm (85% of pixels passing) [2], the dose distributions from all 12 centers meet the criteria using both the RPC and VPP measurement data sets (Table 2). Using more stringent gamma criteria, such as the ±5%/5 mm pass at the 90% pixel level, the RPC had one dataset not meet the criteria whereas all VPP datasets continue to meet the criteria. Significant differences in the dosimetry data gamma evaluations for the RPC sagital plane *vs*. the VPP gamma evaluations were observed for the ±3%/3 mm gamma criteria. A statistical trend was observed for the ±7%/4 mm gamma criteria, but no dosimetric significant difference was observed for the ±5%/5 mm gamma criteria (Table 3).

## Discussion

It is of paramount importance that institutions, within cooperative groups, participating in multi-institutional prospective studies, deliver RT *per* protocol and in a consistent and comparable manner, so as not to corrupt the primary end-point of the trial, obscure actual trial outcomes and more importantly to avoid any undue treatment failure and/or radiation-induced toxicity [7]. Several study groups have been cognizant that the introduction of advanced radiation technologies into clinical trials can jeopardize the success of the trial unless the ability of the participating institutions to utilize these advanced technologies in an accurate and consistent manner is evaluated [9,10]. The introduction of a new treatment modality or technology has been shown to be error prone due to human errors and lack of appropriate training. Reduced deviation rates in clinical trials have been observed with various forms of credentialing [2,4,11]. This is particularly relevant for IMRT, for which the participating institutions must show that they have the ability to generate dose distribution conformity to complex target structures with OARs near the target per a protocol requirements [1].

This initial study comparing the gamma evaluations for a single dosimetry plane between the RPC and VPP dosimetry measurements suggests that further investigation is required prior to allowing IMRT credentialing for clinical prospective studies in H&N cancer to be performed using an institutions’ own phantoms whose data are centrally evaluated. Critical issues that still need to be addressed include the inclusion of all of the dosimetry evaluations for the whole credentialing process (e.g., for RPC CDC use both film planes and the TLD results), data submission issues for the VPP, the differences in the measurement analysis techniques between RPC and VPP methods, and method to designate a pass/fail criteria between the RPC phantom CDC and the VPP. The second issue requires that RPC phantom failures be compared to their VPP counterpart to verify whether the institution’s own measurements will not only identify passing CDCs but also failing CDCs. Another issue is that RPC comprehensively assess the whole QA procedure, ranging from the CT calibration, data transfer, dose calculation- and dose delivery-accuracy, which is obviously not the case of the VPP paradigm. Finally, specific assessment criteria appropriate to the dosimetry gamma evaluation analysis must be determined that is specific to the VPP method. One cannot assume that the criteria used by one methodology used for one center for a CDC would be applicable to another center performing a CDC. Using an acceptance criteria of ±3%/3 mm with a pixel pass rate of 90%, the gamma evaluation analysis results obtained with the RPC_PH_ for the sagittal plane indicates a pass rate of 17% while the VPP institution dosimetry gamma analysis using the same criteria indicated a significantly different (75%) pass rate (Table 2). There was however no significant difference when analyzing at the ±5%/5 mm (90% of pixels passing; pass rate of 92% *vs*. 100% for the RPC and VPP, respectively) between the 12 RPC datasets and the 12 VPP datasets for this limited dosimetry analysis and sample size. It is well know that commercial QA systems have unequal sensitivities to detect dose errors. Interestingly, the majority of our RPC_PH_-INST_PH_ discrepant results stemmed from the Delta 4 system (data not shown), which is known to have suboptimal performances for CDC [12]. It is crucial that more data be compiled before any true judgment is made regarding the VPP credentialing process. Credentialing using the RPC phantom includes a much larger dosimetry dataset than evaluated and used in this study and as such, no equivalence between the RPC and VPP CDC methods for the credentialing process can be made at this time.

The question posed by this study is whether the dosimetry comparisons from various TPSs and phantoms used by each EORTC institution (Table 1) can be used to verify IMRT planning and dose delivery for multi-institution clinical trials as compared to the established RPC phantom dosimetry measurement system. Normally, IMRT credentialing is performed using remote auditing tools, including but not limited to cylindrical target-non target structures [13] or standardized anthropomorphic phantoms [1]. These anthropomorphic phantoms, as used by the RPC, provide uniformity in that they are all the exact same design, use the same dosimeters, the dosimeters are all analyzed in the same manner with the same precision, the dosimetry comparison software and registration is the same and a single set of acceptance criteria is used for all irradiations. The phantoms irradiation studies provide an uniform basic end-to-end CDC on which to assure comparability among multiple institutions. However, as an alternate CDC mechanism to the RPC IMRT CDCs, this study investigated in a limited manner the possibility of performing IMRT credentialing using various phantom types using the VPP paradigm by comparing the dose distribution gamma evaluations in a single plane as an initial step. This study has, however, some major limitations and before the VPP paradigm is deemed acceptable for CDC in prospective trials, a more extensive study and dataset are needed to validate the appropriateness of this proposed dosimetry evaluation and credentialing process. First, the limited number of centers in this study prevents any true direct correlation between the RPC_PH_ and INST_PH_ CDC results. More results are warranted and a further comparison of centralized phantom credentialing *vs*. virtual phantom credentialing is needed. Second, the data from 6 out of 18 institutions (33%) could not be analyzed because of data transfer incompatibilities that have to be resolved. In two cases, the data could not be read by the evaluation software. This problem might be solved in new versions of either the measurement software or evaluation software. In 4 cases, the dataset was incomplete. The fact that, in our analysis, a third of the data stemming from EORTC centers could not be analyzed could be considered tantamount to disproving the ability of the VPP to perform IMRT credentialing within the context of prospective trials. Truth to be told, the retrospective design of this analysis put a lot of pressure on medical physicists from EORTC centers to obtain these data that were neither supportive of a compulsory credentialing requirement process nor did benefit patients treated in busy radiation oncology departments. This problem could be solved with the introduction of prospective credentialing as is performed for NCI funded clinical trials, such that without providing the full dataset, trial participation is not granted. The VPP methodology will be prospectively tested in the new EORTC lung cancer study ( http://www.eortc.org/research-groups/radiation-oncology-group/recent-achievements). Third, in this study only homogeneous VPP phantoms were used and as such, the TPS’s ability to correctly calculate dose in an inhomogeneous geometry was not tested. However, one can require institutions to incorporate additional heterogeneities in the phantom design within a VPP procedure if desired. Finally, the measurement precision of each institution with respect to their phantom and measurement procedure is not known and can be quite different depending on the dosimetry equipment available and expertise of the staff. This might lead possibly to false negative or false positive credentialing results. While a false negative result can be solved by a new measurement, a false positive result cannot be detected as easily as with a centralized phantom (i.e., RPC credentialing) with a small measurement uncertainty. The need to include credentialing phantom failures and compare those results to the VPP CDCs is necessary to ascertain the validity of the VPP process. Credentialing through a VPP should thus never be performed as the only QART check within a trial. Beam Output Audits should always be required, as it is currently for most institutions participating in clinical trials.

This study is essentially the first step in a proof of concept and feasibility study to perform virtual credentialing, however much work is still to be done. The use of standardized anthropomorphic phantoms has undeniably advantages, when compared to the use of institutional phantoms only. The uniformity and quality of data that are collected centrally is unquestionable. This procedure is a comprehensive test of a center’s ability to image, plan and treat a patient. The credentialing procedure has been routinely performed for over a decade and has provided a contemporary benchmark that future procedures cannot exceed; rather, alternative credentialing procedures should try to attain the same level of accuracy, at decreased procedural costs or time. There is the need to continually evaluate this particular credentialing process to determine if there are more efficient methods.

Depending on the specific protocol and its requirements for treatment delivery, the method of credentialing should be identified that best provides the most consistent patient data population for the trial analysis. More specifically, errors in TPS software’s inadequate modeling of the penumbra at MLC leaf ends or inadequate QA of multileaf collimator should be verified. These potential errors could potentially be identified also by institutional credentialing, however the data from > 1000 IMRT H&N phantom irradiations suggest that this is not the case since nearly 20% of the institutions failed the RPC phantom irradiation test using a generous criteria [3]. Incorrect output factors and percent depth dose data, entered by the institution, cannot however be thoroughly verified by an institutional credentialing process without central supervision and absolute dosimetry measurements. Moreover, incorrect systematic application of QA calculations or measurements can be diligently identified by the RPC’s independent audit, but not by the institution itself. These types of dosimetric violations should be routinely identified by the local QA process of participating institutions which have their own QA programs, but because of human error, workload, to name a few, many of these dosimetric discrepancies may go undetected. The RPC’s end-to-end phantom CDC also has drawbacks in that it is difficult to identify the specific component in the chain of events that causes the irradiation failure since it is a composite QA check. As such, the need for an institution to have a rigorous QA program to identify to verify individual dosimetric components of an RT delivery is paramount, but each institution should also have a method, such as an independent QA check, to verify the whole delivery process.

Noteworthy, RPC_PH_ and INST_PH_ CDCs are 2D/absolute and relative dosimetry measurement verification systems, respectively. As such, the measurements from these systems represent only a sparse sampling of the complete 3D treatment volume. QA standards for IMRT QA could be improved by incorporating 3D dosimetry techniques. Sakhalkar et al. reported on the feasibility of relative 3D dosimetry using the RPC phantom for credential testing [14]. Using a 98% pixel level at the ±4%/3 mm distance to agreement in the axial central axis plane, an excellent agreement was observed between the 3D and 2D gamma maps inter-comparison. Both the phantoms and VPP CDCs could be further refined using 3D gamma maps and incorporated within the credentialing program of future prospective trials to provide an improved assessment of an institution’s ability to deliver radiotherapy doses accurately and consistently. Also, incorporation of e.g. breathing motion and heterogeneities in the credentialing procedure also adds to the complexity of the overall credentialing process, as it requires substantial skill’s analysis to handle these phantoms. In contrast, centers in Europe that have introduced treatment techniques incorporating gating or tracking motion management generally have acquired specific QA phantoms for this. Thus, the use of a VPP could potentially also allow a faster credentialing and introduction of these techniques in EORTC clinical trials if the VPP can be made to be uniform in its data collection and assessment. Moreover, these phantoms might also be used to test the 4D CT imaging capabilities of these institutions [15].

In conclusion, IMRT dosimetry gamma evaluations for a single plane of data evaluation for a H&N prospective trial using the institutional phantom measurements showed agreement at the gamma index criteria of ±5%/5 mm (90% of pixels passing) in this analysis that involved a limited number of VPP measurements. Gamma indexes also showed agreement between RPC and VPP metrics at the ±7%/4 mm (85% of pixels passing) but more VPP data sets are needed before a definite conclusion can be reached since we cannot assume that the critieria levels being used by the two evaluations, RPC and VPP, would be the same as the means to compare the two techniques.

## Abbreviations

RT: Radiation therapy; NCI: National Cancer Institute; H&N: Head and Neck; IMRT: Intensity-modulated radiotherapy; RPC: Radiological Physics Center; RPC_PH_: RPC Phantom; INST_PH_: Institutional phantom; VPP: Virtual phantom project; eortc: European Organization for the Research and Treatment of Cancer; CDC: Complex dosimetry checks; QART: Quality assurance in radiotherapy; PTV: Planning Target Volume; TPS: Treatment planning system.

## Competing interests

The authors declare that they have no competing interests.

## Authors’ contributions

DCW and CWH were responsible for the primary concept and the design of the study; CM, VT and VV, performed the data capture and analysis. DCW and CWH drafted the manuscript; DCW performed the statistical analysis; AM, DSF, VV, CWH and DCW reviewed patient data; all authors revised the manuscript. All authors have read and approved the final manuscript.

## Supplementary Material

Additional file 1 List of EORTC institution participating to the Dummy Run for the Phase III trial EORTC 22071-24071. Click here for file
