# Supplementary material for: IMRT credentialing for prospective trials using institutional virtual phantoms: results of a joint European Organization for the Research and Treatment of Cancer and Radiological Physics Center project
Source: Radiat Oncol. 2014 May 29;9:123. doi: 10.1186/1748-717X-9-123 (PMC4046849; doi:10.1186/1748-717X-9-123)
Supplement: Additional file 1 — List of EORTC institution participating to the Dummy Run for the Phase III trial EORTC 22071-24071. [file 1748-717X-9-123-S1.docx]

Additional file 1: List of EORTC institution participating to the Dummy Run for the Phase III trial EORTC 22071-24071

| **EORTC Site Number** | **City** | **Name hospital** | **Country** |
| --- | --- | --- | --- |
|  |  |  |  |
| 457 | Lausanne | CHUV | CH |
| 459 | Bellinzona | Oncology Institute of Southern Switzerland | CH |
| 451 | Geneva | Geneva University Hospitals, Campus Cluse-Roseraie | CH |
| 229 | Dijon | Centre Georges-Francois Leclerc | FR |
| 704 | Milan | Fondazione IRCCS Instituto Nazionale dei Tumori | IT |
| 938 | Pamplona | Hospital de Navarra | ES |
| 1201 | Namur | Clinique Sinte Elisabeth | BE |
| 304 | Nijmegen | Radboud University Nijmwegen Medical Centre | NL |
| 5800 | Poznan | Greaterpoland Cancer Center | PO |
| 101/131 | Brussels | Institut Jules Bordet | BE |
| 308 | Amsterdam | VU University Medical Center | NL |
| 456 | Zürich | UniSpital Zürich | CH |
| 121 | Brussels | Cliniques Universitaires St. Luc | BE |
| 250 | Nancy | Institut de cancerologie de Lorreine Alexis Vautrin | FR |
| 1234 | Wilrijk | Oncologisch Centrum GZA ziekenhuizen | BE |
| 335 | Groningen | University Medical Center Groningen | NL |
| 931 | Haifa | Ramdam Medical Center | IS |
| 147 | Leuven | Universitair ziekenhuis Gasthuisberg Leuven | BE |
